# The Effects of Season of Birth on the Inflammatory Response to Psychological Stress in Hainan Island, China

**DOI:** 10.1371/journal.pone.0139602

**Published:** 2015-10-08

**Authors:** Aki Yazawa, Yosuke Inoue, Andrew Stickley, Dandan Li, Jianwei Du, Chiho Watanabe

**Affiliations:** 1 Department of Human Ecology, Graduate School of Medicine, the University of Tokyo, Bunkyo, Tokyo, Japan; 2 The Stockholm Center for Health and Social Change (Scohost), Södertörn University, Huddinge, Stockholm, Sweden; 3 Hainan Center for Disease Control and Prevention, Haikou, Hainan, China; The University of Kansas Medical Center, UNITED STATES

## Abstract

Season of birth (SOB) has been investigated as one of the environmental factors that might epigenetically determine the physiology of individuals. This study investigated the role of SOB in the association between Quality of Life (QOL), a proxy of psychological stress status, and C-reactive protein (CRP) concentration (i.e., inflammatory status) among 1,085 adults (aged 20–57 years old) in Hainan Island, China. High sensitivity CRP concentration was measured in dried blood spot samples, while the abbreviated version of the World Health Organization’s QOL questionnaire was used to gather information on six QOL domains. Analysis stratified by three historically distinct age groups revealed a significant association between CRP concentration, SOB, QOL and an interaction between SOB and QOL among the youngest and oldest groups. In the oldest group, those born in the dry season had a higher CRP concentration with worse QOL whereas in the youngest group, there was a higher CRP concentration with better QOL. Annual per capita rice production, a proxy of population nutritional status in the year of birth, was found to predict CRP concentration only among the second oldest group. These findings suggest that the early environment might affect the immune response to psychological stress in adulthood and that its effect may differ by the time period in which people were born.

## Introduction

Research has shown that the early life environment (i.e., in the fetal period, infancy and childhood) can epigenetically determine the physiology of individuals and have a life-long effect on health [1]. Season of birth (SOB) is one of the environmental factors that has been previously investigated as it can reflect ecological differences in conditions in early life, such as temperature, precipitation and humidity, and thus, pathogen exposure or experience of food shortage that can have a substantial effect on human physiological development [2].

Evidence suggests that the effects of SOB can manifest themselves across the life course. In a recent review of epidemiological evidence on seasonality of birth outcomes and the impact of climate, Strand et al. [3] identified 20 studies that reported seasonality in birth outcomes, namely preterm birth, stillbirth and low birth weight, and reported that extreme temperature (both low and high temperature) was associated with these undesirable birth outcomes, which have themselves been linked with future chronic diseases like hypertension, diabetes and obesity [4]. Among adults, differences in SOB have been linked to variations in both health behaviors (e.g., smoking) [5] and health outcomes (body mass index [BMI], chronic diseases, suicide, mortality and longevity) [6–9].

Although the physiological mechanisms underlying the association between SOB and health are uncertain, research by McDade et al. [10] has focused on differences in C-reactive protein (CRP) concentration, which is a plasma protein involved in the systemic response to inflammation, and regarded as a risk marker for future chronic diseases [11,12], as a possible factor linking SOB and health outcomes. In a study of young adults aged 20–22 years old in the Philippines, they showed that CRP concentration was influenced by SOB. More specifically, the association between psychological stress (as indexed by the Perceived Stress Scale) and CRP concentration was positive among those who were born in the ‘not dry season’, whereas no such association was observed among those born in the dry season. This led them to highlight the importance of nutritional and microbial exposure in infancy as potential factors that differentiate physiological development by SOB [10]. Their findings also suggest that the effects of SOB might be manifested in the form of a physiological response to psychological stressors.

The current study was designed to build on and extend the earlier study by McDade and colleagues in several ways. First, the current study was undertaken in China—a country which experienced especially dramatic societal changes during the second half of the twentieth century. Given the unique social, economic and political events that occurred during that period, and the possibility that they may have impacted on the early life environment in different ways (e.g., through improvement in agricultural technology [13]), we examined the SOB—CRP association for participants born in three distinct historical epochs: (i) after World War II to the end of the Great Leap Forward (1953–1962), (ii) the Cultural Revolution (1963–1977) and (iii) the period of early economic reforms (1978–1990) [14,15]. Second, it is also possible that inter-year variation in population nutritional status within each epoch further affected the SOB—CRP association. Indeed, there is evidence that food production and availability fluctuated greatly across the period [16], to the point where there was even famine (in 1958 to 1962) [14]. Thus, to examine the effects of early life nutritional status on inflammatory response in adulthood we used data on per capita annual rice production (available from 1953 to 1990 [16]) as a proxy measure for population nutritional status in the year when people were born. Third, this historical focus enabled us to use data from participants with a much broader age range than had been previously examined.

The specific aims of this study were therefore to determine whether SOB affects the inflammatory reaction (indexed as CRP concentration) to psychological stressors (as measured by the Quality of Life [QOL] score) among adults and whether there are differences in these relations between different age groups, or in relation to the availability of food during the participants’ formative years.

## Methods

### Study location

**Fig 1:**
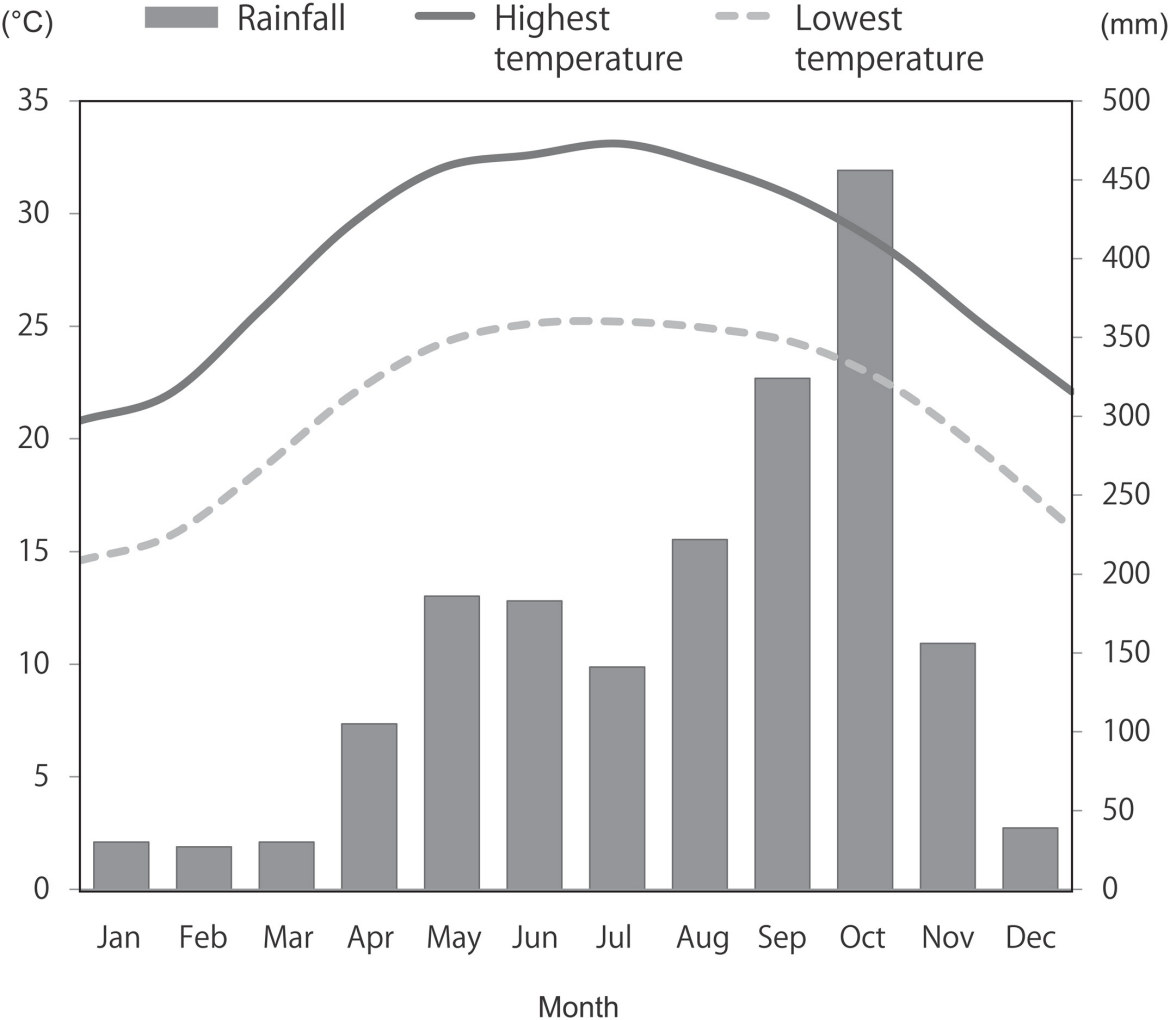
Average monthly temperature and rainfall in Hainan Island, China (2000–2012). Data source: World Weater Online.

This study used data from Hainan Island, China, which is located to the south of mainland China. It has a land area of 33,210 km^2^ with a subtropical monsoon climate with two distinct seasons (i.e., a rainy season and a dry season), an annual average temperature ranging from 18°C to 25°C and annual precipitation ranging from 1,500 mm to 2,000 mm. For statistical purposes, in this study the period between May to October was defined as the rainy season and the other six months of the year as the dry season. This division was based on monthly precipitation data: the average monthly precipitation was 252.0 mm for the rainy season and 64.5 mm for the dry season [17] (see Fig 1).

In 1949, the People’s Republic of China was established after the Chinese Civil War. From that point until the beginning of the 1990s, there were 3 distinct historical periods. The ‘Great Leap Forward’, which lasted until the early 1960s was marked by land reform and ongoing revolution and culminated in a severe famine (the Great Famine) [14]. This was followed by the period of ‘Cultural Revolution’ which was notable for political confusion, unrest and repression. At the end of the 1970s a new and distinct historical phase began with the introduction of a market economy (economic reform) in 1978 [15]. Although the economy in Hainan Island remained comparatively underdeveloped for a longer period of time when compared with other parts of the country, residents also experienced pronounced changes in their living environment which gathered pace after 1990 [18,19].

### Field survey and biomarker data collection

This study was undertaken as part of a collaborative research project between the University of Tokyo and Hainan Provincial Center for Disease Control and Prevention, China. To recruit participants, convenience sampling was conducted in 21 communities in 6 different regions, which encompassed the range of economic development across the island. Questionnaire data and dried blood spot (DBS) samples were collected in November to December, 2010. All of the people residing in the selected study communities were invited to participate in the study. Interviews were conducted by trained fieldworkers with information being obtained on a variety of demographic and socioeconomic indicators including age (in years), date of birth, sex, BMI, educational attainment (schooling of less than 9 years; equivalent to 9 years; and more than 9 years) and marital status (currently married; and not currently married). The current study involved participants who were born in the period between 1953 and 1990 when China experienced rapid, large-scale change in all aspects of life (e.g., income levels, hygienic conditions, energy intake, dietary composition and so on [13]), i.e., those who were aged 20–57 years old as of 2010. Those who did not provide information on their month of birth (n = 336) were excluded from the analysis.

The abbreviated version of the World Health Organization’s Quality of Life questionnaire (WHOQOL-BREF) (Chinese version) [20] was used to evaluate subjective QOL of the respondents. The WHOQOL-BREF comprises 26 items that encompass a number of different aspects of life, all of which are scored using a five-point Likert scale, with higher scores indicating a better QOL. The first two items evaluate overall QOL and general health, whereas the remaining 24 items are divided into four QOL domains: *physical* domain, *psychological* domain, *social relationship* domain and *environment* domain. The six subscale scores were dichotomized at the median in the statistical analysis in order to make our results more comparable with those from McDade et al. [10] where Perceived Stress Scale scores were dichotomized when assessing psychological stress.

DBS samples were used for the measurement of CRP concentration. A tiny amount of whole blood was directly collected on quantitative filter paper (903 Protein Saver Cards, Whatman Kent, UK). Duplicated DBS samples were assayed for CRP concentration with a high-sensitivity sandwich enzyme immunoassay (EIA) following a previously established protocol [21]. If a participant’s serum-equivalent CRP concentration exceeded 10 mg/L, then they were judged as experiencing inflammation due to acute infection [22], and were excluded from the analysis.

### Statistical analysis

A least-squares regression analysis was used to investigate the association between log-transformed CRP concentration and each QOL domain score. As the WHOQOL-BREF provides a profile of scores across six different aspects of QOL we followed the lead of several previous studies that have focused separately on the individual domains [23–25]. Statistical analyses were initially undertaken including all participants. Subsequent analyses were then performed separately for the three historical age groups (20–32, 33–47, 48–57 years old) to examine whether any observed associations differed between them. Each model included an interaction term for the QOL score and SOB to examine whether the association between the QOL scores and CRP concentration might be modified by SOB, that is, the early life environment. In addition, data on annual per capita rice production were also included in each statistical model. The rice production data was obtained from a (Chinese) publication entitled, ‘A History of Hainan Province—Food History’ [16] and reported in terms of yearly gross production (kg). The annual population of the island was used as the denominator when calculating yearly per capita production. Other covariates included in all analyses were age (a continuous variable in years), sex, BMI (kg/m^2^), educational attainment (schooling of less than 9 years; equivalent to 9 years; and more than 9 years), and marital status (currently married; and not currently married).

All statistical analyses were conducted using Stata version 13.1 (StataCorp, College Station, TX). The level of statistical significance was set at *p* < 0.05. The data used in the analyses is provided in Supporting Information (S1 Dataset).

### Ethical approval

The study protocol was approved by the Research Ethics Committee at the Graduate School of Medicine, University of Tokyo (ethnical approval No. 3406) and Hainan Provincial Center for Disease Control and Prevention. Written informed consent was obtained from all study participants before they were enrolled into the study.

## Results

### Characteristics of the study participants

Characteristics of the study participants are shown in Table 1. Among the 1,085 participants included in the analysis, 47.1% were male. The mean age of the participants was 38.1 years (standard deviation [SD] 10.3), while they had a mean BMI of 22.3 (SD 3.2). Forty-four percent of the study subjects were born in the dry season. The median CRP concentration (inter-quartile range) was 0.73 (0.33–1.85) mg/L. The mean overall QOL score was 2.9 (SD 0.7), while the scores in the other domains were: general health 3.2 (SD 0.9), *physical* domain 3.5 (SD 0.5), *psychological* domain 3.1 (SD 0.6), *social relationship* domain 3.6 (SD 0.5) and *environment* domain 2.9 (SD 0.5).

**Table 1:** Characteristics of included and excluded participants (aged 20–57 years old).

Variables	Participants (N = 1,085)		Excluded (n = 336)		*P* ^b^
	Mean [SD] / n (%)		Mean [SD] / n (%)		
Age (years)	38.1	[10.3]	42.0	[9.1]	<0.001
Sex					0.91
Male	511	(47.1)	157	(46.7)	
Female	574	(52.9)	179	(53.3)	
BMI (kg/m^2^)	22.3	[3.2]	21.5	[3.1]	<0.001
Education					<0.001
Less than 9 years	599	(30.8)	218	(64.9)	
9 years	570	(52.5)	107	(31.8)	
More than 9 years	116	(10.7)	11	(3.3)	
Marriage (Married)	933	(86.0)	288	(85.7)	0.90
Season of birth (Dry season)	478	(44.1)	-	-	
Quality of Life (QOL)^a^					
Overall QOL	2.9	[0.73]	2.8	[0.82]	0.007
General Health	3.2	[0.89]	3.0	[0.93]	<0.001
*Physical* domain	3.5	[0.53]	3.3	[0.53]	<0.001
*Psychological* domain	3.1	[0.56]	3.1	[0.62]	0.22
*Social relationship* domain	3.6	[0.52]	3.6	[0.51]	0.31
*Environment* domain	2.9	[0.52]	2.8	[0.45]	0.007
C-reactive protein (mg/L)^c^	0.73	[0.33–1.85]	0.59	[0.27–1.26]	0.02

^a^: Quality of Life was scored by WHOQOL-BREF.

^b^: Student’s t-test for continuous values and Pearson’s chi-square test for categorical values were conducted to examine if there were differences for variable scores between the participants and those who were excluded.

^c^: Median and inter-quartile range is shown for C-reactive protein concentration.

### Relationship between CRP concentration, QOL scores, SOB and annual per capita rice production

Table 2 shows the results of a least-squares regression analysis investigating the association between CRP concentration, the QOL score for each domain, SOB and annual per capita rice production among all of the participants. None of the QOL domain scores were associated with CRP concentration. Similarly, SOB was not associated with CRP concentration in any of the models. Further examination of these variables using an interaction term for QOL and SOB also produced no significant associations, while annual per capita rice production was also not associated with CRP concentration in any model. In terms of the control variables age (a continuous variable in years) was not associated with CRP concentration, while BMI was positively associated with CRP concentration in all models, as was being male (data not shown). Neither educational attainment nor marital status associated with CRP concentration (data not shown).

**Table 2:** The association between log-transformed CRP concentration and QOL scores, SOB and rice production among participants aged 20–57 in Hainan Island, China in 2010 (N = 1,085).

	Model 1		Model 2		Model 3		Model 4		Model 5		Model 6	
	B	*p*	B	*p*	B	*p*	B	*p*	B	*p*	B	*p*
Quality of Life (QOL)												
Overall QOL	0.033	0.54										
General health			−0.007	0.89								
*Physical* domain					0.023	0.61						
*Psychological* domain							0.030	0.51				
*Social relationship* domain									0.085	0.053		
*Environment* domain											0.066	0.13
Season of birth (SOB) (ref. Dry season) Rainy season	−0.085	0.18	−0.067	0.27	−0.062	0.19	−0.027	0.58	−0.022	0.62	−0.015	0.74
QOL x SOB	0.095	0.18	0.073	0.29	0.084	0.17	0.024	0.69	0.017	0.77	0.006	0.92
Rice production [Yearly per capita gross production (kg)]	<0.001	0.38	<0.001	0.36	−0.001	0.27	<0.001	0.34	<0.001	0.39	<0.001	0.35
R-squared	0.107		0.102		0.107		0.102		0.109		0.105	

The least-squares regression models were adjusted for age (a continuous variable in years), sex, body mass index (kg/m^2^), education level (less than 9 years; 9 years; and more than 9 years) and marital status (currently married; and not currently married). Models 1–6 include the following QOL scores, respectively: Overall QOL (Model 1), General health (Model 2), *Physical* domain (Model 3), *Psychological* domain (Model 4), *Social relationship* domain (Model 5), and *Environment* domain (Model 6). Rice production is shown as yearly per capita gross production (kg).

Analyses that were conducted for the three age groups (i.e., 20–32, 33–47, 48–57 years old) revealed that QOL was positively associated with CRP concentration among participants aged 20–32 year old for the *environment* domain (coefficient = 0.200, *p* = .013) (Table 3) and among those aged 33–47 years old for the *physical* domain (coefficient = 0.163, *p* = .018) (Table 4). There were significant negative associations among participants aged 48–57 years old for overall QOL (coefficient = − 0.197, *p* = .042), for general health (coefficient = − 0.417, *p* < .001) and for the *physical* domain (coefficient = − 0.241, *p* = .004) (Table 5). For those who were born in the rainy season among the oldest age group (48–57), overall QOL (coefficient = − 0.249, *p* = .027), general health (coefficient = − 0.369, *p* = .002), *physical* domain (coefficient = − 0.266, *p* = .002) and *environment* domain (coefficient = − 0.204, *p* = .023) were all associated with a lower CRP concentration (Table 5). There was no statistically significant association between CRP concentration and the interaction between each QOL score and SOB among participants aged 20–32 and 33–47 years old, except for the model with *environment* domain among participants aged 20–32 (coefficient = − 0.227, *p* = .035) (Table 3). A statistically significant interaction between QOL and SOB was observed among participants aged 48–57 years old for overall QOL (coefficient = 0.331, *p* = .011), general health (coefficient = 0.465, *p* = .001), *physical* domain (coefficient = 0.464, *p* < .001), and *environment* domain (coefficient = 0.358, *p* = .003) (Table 5).

**Table 3:** The association between log-transformed CRP concentration and QOL scores, SOB and rice production among participants aged 20–32 years old in Hainan Island, China in 2010 (n = 378).

	Model 1		Model 2		Model 3		Model 4		Model 5		Model 6	
	B	*p*	B	*p*	B	*p*	B	*p*	B	*p*	B	*p*
Quality of Life (QOL)												
Overall QOL	0.213	0.052										
General health			0.147	0.15								
*Physical* domain					0.071	0.38						
*Psychological* domain							0.036	0.69				
*Social relationship* domain									0.087	0.28		
*Environment* domain											0.200	0.013
Season of birth (SOB) (ref. Dry season) Rainy season	0.088	0.50	0.125	0.30	<0.001	0.99	−0.032	0.74	−0.038	0.62	0.072	0.37
QOL x SOB	−0.164	0.25	−0.220	0.10	−0.099	0.36	−0.027	0.82	−0.019	0.86	−0.227	0.035
Rice production [Yearly per capita gross production (kg)]	<0.001	0.89	<0.001	0.99	<0.001	0.90	<0.001	0.93	<0.001	0.91	<0.001	0.86
R-squared	0.108		0.105		0.101		0.099		0.103		0.114	

The least-squares regression models were adjusted for age (a continuous variable in years), sex, body mass index (kg/m^2^), education level (less than 9 years; 9 years; and more than 9 years) and marital status (currently married; and not currently married). Models 1–6 include the following QOL scores, respectively: Overall QOL (Model 1), General health (Model 2), *Physical* domain (Model 3), *Psychological* domain (Model 4), *Social relationship* domain (Model 5), and *Environment* domain (Model 6). Rice production is shown as yearly per capita gross production (kg).

**Table 4:** The association between log-transformed CRP concentration and QOL scores, SOB and rice production among the participants aged 33–47 years old in Hainan Island, China in 2010 (n = 477).

	Model 1		Model 2		Model 3		Model 4		Model 5		Model 6	
	B	*p*	B	*p*	B	*p*	B	*p*	B	*p*	B	*p*
Quality of Life (QOL)												
Overall QOL	0.056	0.48										
General health			0.059	0.44								
*Physical* domain					0.163	0.018						
*Psychological* domain							0.069	0.31				
*Social relationship* domain									0.086	0.19		
*Environment* domain											0.076	0.25
Season of birth (SOB) (ref. Dry season) Rainy season	−0.061	0.50	−0.059	0.50	0.043	0.55	0.040	0.57	<0.001	>0.99	0.016	0.81
QOL x SOB	0.116	0.26	0.114	0.26	−0.018	0.84	−0.025	0.78	0.039	0.66	0.015	0.86
Rice production [Yearly per capita gross production (kg)]	−0.003	0.049	−0.003	0.038	−0.003	0.021	−0.003	0.032	−0.003	0.045	−0.003	0.033
R-squared	0.128		0.129		0.136		0.118		0.127		0.123	

The least-squares regression models were adjusted for age (a continuous variable in years), sex, body mass index (kg/m^2^), education level (less than 9 years; 9 years; and more than 9 years) and marital status (currently married; and not currently married). Models 1–6 include the following QOL scores, respectively: Overall QOL (Model 1), General health (Model 2), *Physical* domain (Model 3), *Psychological* domain (Model 4), *Social relationship* domain (Model 5), and *Environment* domain (Model 6). Rice production is shown as yearly per capita gross production (kg).

**Table 5:** The association between log-transformed CRP concentration and QOL scores, SOB and rice production among participants aged 48–57 years old in Hainan Island, China in 2010 (n = 230).

	Model 1		Model 2		Model 3		Model 4		Model 5		Model 6	
	B	*p*	B	*p*	B	*p*	B	*p*	B	*p*	B	*p*
Quality of Life (QOL)												
Overall QOL	−0.197	0.042										
General health			−0.417	<0.001								
*Physical* domain					−0.241	0.004						
*Psychological* domain							−0.049	0.58				
*Social relationship* domain									0.150	0.083		
*Environment* domain											−0.147	0.10
Season of birth (SOB) (ref. Dry season) Rainy season	−0.249	0.027	−0.369	0.002	−0.266	0.002	−0.142	0.12	0.005	0.95	−0.204	0.023
QOL x SOB	0.331	0.011	0.465	0.001	0.464	<0.001	0.229	0.051	−0.014	0.90	0.358	0.003
Rice production [Yearly per capita gross production (kg)]	<0.001	0.64	0.001	0.47	0.001	0.56	<0.001	0.63	<0.001	0.62	0.001	0.53
R-squared	0.127		0.165		0.167		0.124		0.125		0.141	

The least-squares regression models were adjusted for age (a continuous variable in years), sex, body mass index (kg/m^2^), education level (less than 9 years; 9 years; and more than 9 years) and marital status (currently married; and not currently married). Models 1–6 include the following QOL scores, respectively: Overall QOL (Model 1), General health (Model 2), *Physical* domain (Model 3), *Psychological* domain (Model 4), *Social relationship* domain (Model 5), and *Environment* domain (Model 6). Rice production is shown as yearly per capita gross production (kg).

Rice production was significantly inversely associated with CRP concentration in all of the models among participants aged 33–47 years old (Table 4), while it was not associated with CRP concentration in any models among the other two age groups.

**Fig 2:**
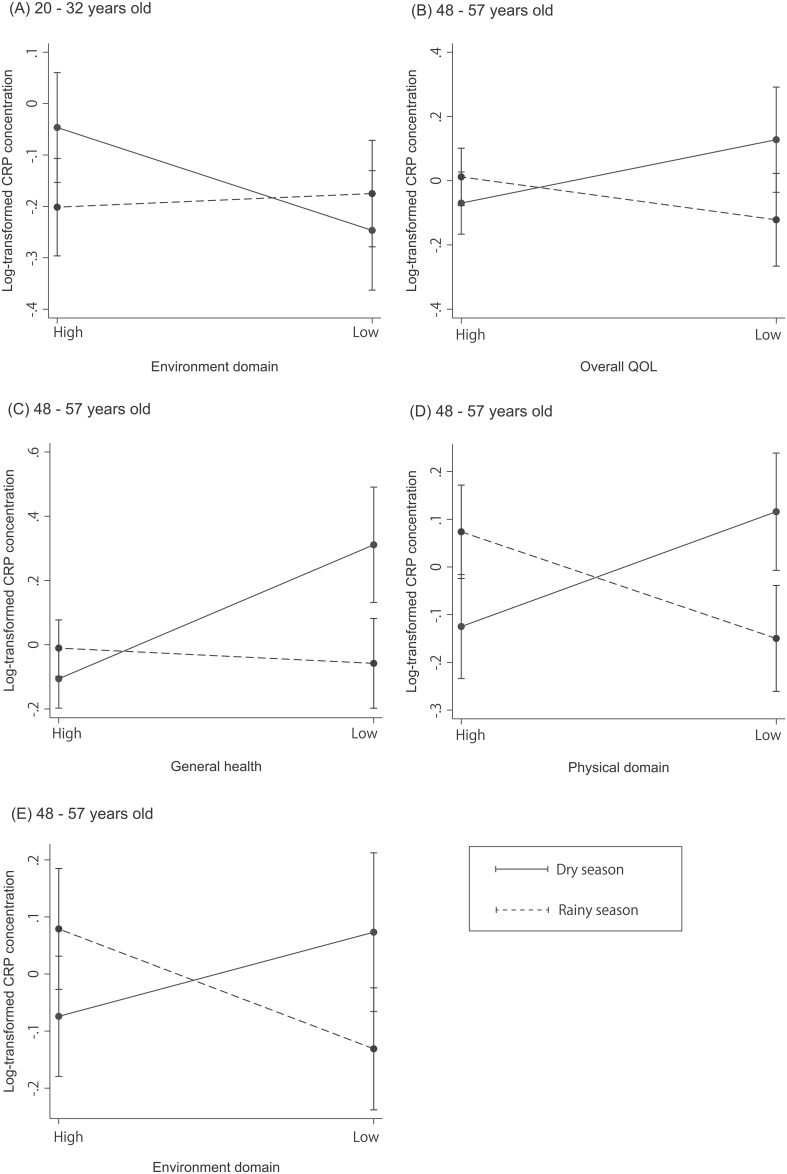
The association between season of birth (SOB), Quality of Life (QOL) and C-reactive protein (CRP) concentration.

Fig 2A–2E illustrates predicted CRP concentration for SOB and QOL after adjusting for variables included in the models where a statistically significant interaction was detected between QOL and SOB. Among the oldest age group, people who were born in the dry season had increased CRP concentration with a higher level of stress (i.e., as indicated by a lower QOL in terms of overall QOL, general health, and in the *physical* domain) (Fig 2B–2D), while people who were born in the rainy season had decreased CRP concentration when their stress level was higher (i.e., they had a lower QOL in *physical* and *environment* domains) (Fig 2D and 2E). On the other hand, among the youngest age group, people who were born in the dry season had decreased CRP concentration when having lower *environment* domain QOL (Fig 2A).

## Discussion

### Summary of findings

Analyses conducted separately by age group revealed that the inflammatory reaction (indexed as CRP concentration) to psychological stress (as indicated by a lower QOL score) in adulthood differed by SOB. Specifically, we showed that SOB modified the inflammatory response to psychological stress among people aged 48–57 years old (four QOL subscale scores) and people aged 20–32 years old (one QOL subscale score). Annual per capita rice production modified adult inflammatory reaction only among people aged 33–47 years old. These associations were masked when analyzing all ages combined.

### Mechanisms linking SOB and CRP concentration in adulthood

Studies have suggested that two mechanisms might help explain the role of SOB in determining adult inflammatory response to psychological stress: microbial exposure in infancy and nutritional status in utero. For the former, research has highlighted the important role that seasonality can have in relation to the spread of pathogens. This might be related in part, to seasonal differences in weather conditions. In particular, it seems that pathogens often become more widespread after heavy rain or flooding [26]. Moe et al. [27] and VanDerslice et al. [28] have both shown the important effect that rainfall has on the spread of pathogens in the Philippines. Other research has highlighted the role of temperature. Xu et al. [29] for example, examined the seasonal pattern of childhood diarrhea in Brisbane, Australia, and concluded that both low and high temperatures increased its incidence. Chen et al. [30] also demonstrated the seasonality of common respiratory virus activities in a subtropical area in China when investigating the association between these viruses and temperature, humidity and rainfall. As pathogen exposure during early childhood has been linked to the development of anti-pathogen defenses in adulthood [31], this research suggests that differences in seasonal-mediated pathogen exposure might play a role in determining variations in the physiology of individuals.

Regarding in utero nutritional status, in a recent review study, Chodick et al. [32] showed that in several developing countries with a tropical / subtropical climate (e.g., Zaire, India and Taiwan), birth weight tends to be highest in the beginning of the dry season and lowest in the beginning of the rainy season. They interpreted this pattern as a result of maximal fetal growth in the third trimester during the harvesting season which is towards the end of the rainy season. As low birth weight has been associated with immature development of the immune system, which can lead to higher CRP in children and adults [10,33], the better nutritional status in utero of those who were born in the end of the rainy season might have resulted in a lower CRP concentration in adulthood.

### The relationship between SOB and CRP concentration in Hainan Island

In the current study, the relationship between SOB and CRP concentration among the oldest age group differed from that observed in a recent study by McDade et al. [10]. Specifically, people who were born in the dry season in Hainan had an elevated inflammatory status when they were psychologically stressed compared to those who were born in the rainy season. In contrast, in McDade et al.’s study, people born in the dry season had a lower inflammatory status when exposed to psychological stress compared to those born in the rainy season.

When trying to understand the seasonal difference in the development of immune function (i.e., inflammatory response in adulthood) between McDade’s study and this study, it should be borne in mind that there are important differences between the study locations (i.e., Hainan and Cebu). First, in Hainan, the Lunar New Year is an important event. This is a period associated with food-intensive celebrations lasting for two weeks during the period from the end of January to the beginning of February. In terms of our results, it can be speculated that during the time of the Great Leap Forward, the impact of such an occasion might have been especially large, particularly in contrast to the lack of food possibly consumed at other times in the year. Therefore, those who were born in the rainy season may have had a richer nutritional intake in utero. Sufficient nutritional intake in utero contributes to higher birth weight which, in turn, might result in lower CRP production in the presence of stressors [34]. This might explain why among those who were older, immune function was regulated for those born in the rainy season even when stressed but why CRP concentration was higher for those born in the dry season when similarly stressed.

Second, the seasonal variation in temperature is larger in Hainan than in Cebu: the mean monthly maximum temperatures during the dry and rainy season are 22.8°C and 28.3°C in Hainan but 31.0°C and 31.4°C in Cebu, respectively [17,35]. Temperature change can stimulate the immune system [36]. It is possible therefore that Hainan’s larger temperature range between seasons might put those who are born in the early dry season at greater risk of developing a phenotype with a higher immunological reaction to psychological stress, which again might help explain why those who were born in the dry season among the oldest age group had a higher CRP concentration when psychologically stressed.

Third, the duration of the dry season is longer in Hainan. In their study, McDade et al. [10] suggested that early infant experience may be critical for the development of the immune system and that compared to people who were born outside of the dry season, individuals who were born in the dry season, that lasts from February to April, might be at higher risk of early post-natal exposure to pathogens during the rainy season. However, as the duration of the dry season is longer in Hainan than in Cebu, those who were born in the early dry season may not experience the same risk of post-natal exposure to pathogens during the rainy season. This might have led to smaller differences in pathogen exposure between those who were born in the rainy season and the dry season in Hainan, compared to Cebu. If this supposition is correct, the SOB effect in terms of pathogen exposure and its subsequent effects might have differed between the two locations.

The direction of the interaction effect observed for the youngest age group in this study mirrored that in McDade et al. [10]. However, we saw a significant decrease in CRP concentration with decreased QOL among those born in the dry season (Fig 2A), whereas in McDade et al.’s study there was a significant increase in CRP concentration with a higher Perceived Stress Scale score among those born in the rainy season [10]. Given that our results for the older age groups suggest that in Hainan, being born in the rainy season is associated with the development of a better immune function and that those with a dry season birth are more susceptible to the surrounding environment, it can be hypothesized that the negative association observed between CRP concentration and decreased QOL among those young people who were born in the dry season might have resulted from increased affluence as manifest in a higher living standard and richer diet, which can lead to both higher perceived QOL and higher CRP [37]; CRP concentration has been known to be associated with an “urban” lifestyle (such as a rich diet [38–40], reduced physical activity [41] and smoking [40]). In effect, this means that for the younger generation the effects of SOB may be being masked by other more powerful factors that are affecting both QOL and CRP production. This hypothesis should be tested in future studies.

### Change in the ecological determinants of immune development across time

Rice production was significantly inversely associated with CRP concentration in all of the models among participants aged 33–47 years old, who were born during the period of the Cultural Revolution, although it was not associated with CRP concentration in any of the QOL domains among the other two age groups. It is possible that this difference might be related to the changing availability of rice and its importance in the Hainan diet across time. In particular, in contrast to the time of the Great Leap Forward where rice production was much lower and there was comparatively little inter-individual variation in its consumption, the years of the Cultural Revolution were marked by a greater production of rice and thus, greater potential for differences in consumption—especially in an environment marked by social instability and political persecution. Although rice production continued to expand in the latter half of the twentieth century, it is possible that its role in the Chinese diet became comparatively less important as lifestyles (and food consumption patterns) became increasingly diversified against a backdrop of economic growth [18,42,43]. If this is the case, it might explain why there was no ‘rice effect’ observed for the youngest participants.

### Study limitations

This study has several limitations. First, we did not have information on several covariates which were previously tested (e.g., socioeconomic status both at birth and at the time of sampling) [10]. This might have impacted on our ability to examine the effects of SOB on the inflammatory response to psychological stress later in life. Second, we used QOL to measure psychological stress status, while in McDade et al. [10] the Perceived Stress Scale [44] was used. These questionnaires may capture different aspects of psychological stress. However, a negative association between the WHOQOL-BREF scores (*physical* and *psychological* domains) and Epstein-Barr virus antibody titer, which is a well-established biomarker for psychological stress status, has previously been observed in Hainan [45], therefore, it is probable that QOL scores adequately reflect psychological stress status in this location. Third, we used a single measurement of CRP concentration to assess baseline inflammation levels. However, previous studies [21] have also justified the use of single measurements of CRP concentration due to the lack of intra-individual variation. Moreover, omitting those with obvious signs of acute inflammation (i.e., serum-equivalent CRP concentrations of > 10 mg/L) meant that we were able to evaluate baseline CRP concentration in a more appropriate way. It is also important to acknowledge that CRP can be a marker for other things besides stress such as ill health, lifestyle factors etc. While we controlled for a number of different variables in the analysis, it is nonetheless possible that the results we obtained may have been subject to unmeasured confounding [46]. Future studies should ensure that all possible variables that can affect CRP are considered when examining its relation with stress. Fourth, this study’s cross-sectional design meant that it was not possible to investigate whether factors such as aging were important for the participants’ physiological response to stressors [47]. However, including age as a continuous variable could have accounted for any possible confounding. Fifth, date of birth was not verified by birth certificates. Sixth, people who were excluded from the analysis were older, had a lower BMI, lower education level and lower CRP concentration than the participants included in this study (Table 1). Also, they had lower QOL scores than the study participants. Therefore, our participants might not be fully representative of all residents in Hainan.

## Conclusion

The findings from this study suggest that inflammatory response to psychological stress in adulthood may differ by season of birth among adults from different age groups in Hainan, China. In addition, population nutritional status (as indexed by per capita annual rice production) was negatively associated with bodily inflammation among the second oldest age group (i.e., 33–47 years). These results seem to accord with those from earlier studies which have shown that the early life environment plays a significant role in determining inflammatory response to psychological stress in adulthood. However, they also highlight the important finding that the ecological factors associated with population physiological development may differ markedly according to the specific context and time period in which people were born.

## Supporting Information

S1 Dataset The data used in the analyses. (CSV)
